# Discovery of novel small molecule modulators of *Clavibacter michiganensis* subsp. *michiganensis*

**DOI:** 10.3389/fmicb.2015.01127

**Published:** 2015-10-19

**Authors:** Xiulan Xu, Anand Kumar, Loïc Deblais, Ruby Pina-Mimbela, Corey Nislow, James R. Fuchs, Sally A. Miller, Gireesh Rajashekara

**Affiliations:** ^1^Department of Plant Pathology, Ohio Agricultural Research and Development Center, The Ohio State UniversityWooster, OH, USA; ^2^Food Animal Health Research Program, Department of Veterinary Preventive Medicine, The Ohio State UniversityWooster, OH, USA; ^3^Pharmaceutical Sciences, University of British ColumbiaVancouver, BC, Canada; ^4^College of Pharmacy, The Ohio State UniversityColumbus, OH, USA

**Keywords:** Cmm, tomato canker, bioluminescent imaging, small molecule inhibitors, high throughput screening

## Abstract

*Clavibacter michiganensis* subsp. *michiganensis* (Cmm) is a Gram-positive seed-transmitted bacterial phytopathogen responsible for substantial economic losses by adversely affecting tomato production worldwide. A high-throughput, cell-based screen was adapted to identify novel small molecule growth inhibitors to serve as leads for future bactericide development. A library of 4,182 compounds known to be bioactive against *Saccharomyces cerevisiae* was selected for primary screening against Cmm wild-type strain C290 for whole-cell growth inhibition. Four hundred sixty-eight molecules (11.2% hit rate) were identified as bacteriocidal or bacteriostatic against Cmm at 200 μM. Seventy-seven candidates were selected based on Golden Triangle analyses for secondary screening. Secondary screens showed that several of these candidates were strain-selective. Several compounds were inhibitory to multiple Cmm strains as well as *Bacillus subtilis*, but not to *Pseudomonas fluorescens, Mitsuaria* sp., *Lysobacter enzymogenes*, *Lactobacillus rhamnosus*, *Bifidobacterium animalis*, or *Escherichia coli*. Most of the compounds were not phytotoxic and did not show overt host toxicity. Using a novel 96-well bioluminescent Cmm seedling infection assay, we assessed effects of selected compounds on pathogen infection. The 12 most potent novel molecules were identified by compiling the scores from all secondary screens combined with the reduction of pathogen infection *in planta*. When tested for ability to develop resistance to the top-12 compounds, no resistant Cmm were recovered, suggesting that the discovered compounds are unlikely to induce resistance. In conclusion, here we report top-12 compounds that provide chemical scaffolds for future Cmm-specific bactericide development.

## Introduction

Tomato bacterial canker, caused by *Clavibacter michiganensis* subsp. *michiganensis* (Cmm), is one of the most important diseases of tomato in temperate zones and greenhouses worldwide (Gleason et al., 1993). Cmm is a Gram-positive bacterium that infects the plant through wounds, and natural openings such as stomata and hydathodes, after which it moves into the xylem (Carlton et al., 1998; Gartemann et al., 2003; Sharabani et al., 2013). Severe yield loss can result from stunting and wilting of the plant, and “bird’s eye” lesions on the fruit. It is known that Cmm is a strong endophyte and is easily disseminated directly into vascular tissue during transplanting, pruning, and harvesting under current tomato production systems (Chang et al., 1991; Kawaguchi et al., 2010). At present, control and management of tomato bacterial canker relies primarily on the use of clean seed, healthy transplant practices, and crop rotation. However, once disease is established in a field or greenhouse, chemical treatment such as copper-based bactericides or antibiotics have a limited impact on reducing the disease burden (Hausbeck et al., 2000; Miller and Ivey, 2005). Cmm control is further complicated by the development of bactericide resistance (Cooskey, 1990). Therefore, novel compounds with new targets are urgently needed for future management of tomato bacterial canker.

High throughput (HTP) screening has proven to be useful in identifying small molecule anti-infectives targeting a specific protein or by inhibiting pathogen (Hong-Geller and Micheva-Viteva, 2013). Small, drug-like molecules (e.g., less than 500 Da with a cLogP less than 5) are particularly attractive because they can often pass through cell membranes. Successful examples include inhibitors to Type III secretion and biofilm formation by *Pseudomonas* (Junker and Clardy, 2007; Arnoldo et al., 2008; Aiello et al., 2010), and novel kinase inhibitors to *Toxoplasma gondii* and acetyl transferase inhibitors in *Escherichia coli* (Pereira et al., 2009; Kamau et al., 2012). Identification of “hits” from such HTP screens can provide the starting point for chemical tools to probe mechanisms of action and for drug development for infectious diseases.

A limited number of studies have used HTP screening in plant-pathogen models. Schreiber et al. (2008) developed a 96-well plate liquid assay to screen small molecules that prevent symptoms caused by *Pseudomonas syringae* on *Arabidopsis thaliana* and uncovered a family of sulfanilamide compounds that reduce bacterial virulence *in planta* (Schreiber et al., 2008). Using a similar approach, further investigation of small molecules targeting the fungal phytopathogen *Fusarium graminearum* identified two compounds, sulfamethoxazole and indole alkaloid gramine, that reduced pathogen infection in wheat (Schreiber et al., 2011). Nonetheless, no studies have been reported that identify small molecules that interact with plant pathogenic Gram-positive bacteria.

In this study we screened a validated library of 4,182 yeast-active molecules or, “yactives” against Cmm by using a whole-cell based HTP screening approach and 77 of the 468 hits were further evaluated for their sensitivity, specificity, and phytotoxicity (Wallace et al., 2011). Candidates were further tested for mammalian cytotoxicity and for Cmm inhibition in tomato seedlings using a bioluminescent Cmm strain (Xu et al., 2010). A structural analysis of the 12 most promising small molecules identified chemical scaffolds for potential bactericide development for future applications.

## Materials and Methods

### Chemical Library and Bacterial Strains

A small molecule library containing 4,182 compounds ‘yactives’ was designed in collaboration with ChemBridge (San Diego, CA, USA) and was supplied in a 96-well format in 10 mM dimethyl sulfoxide (DMSO). We used the ‘yactive’ library because in our previous study it increased hit rate by several folds when screened for model bacteria such as Gram negative *E. coli* (∼12-fold) and Gram positive *Bacillus subtilis* (sixfold) compared to screening random selected compounds library (Wallace et al., 2011). Bacterial strains used in this study are listed in **Table 1**. Bacterial strains were streaked out from -80°C freezer stock onto nutrient broth-yeast agar (NBY) and LB.

**Table 1:** List of bacterial strains tested in the primary and secondary screens.

Bacteria	Strain	Reference
*Clavibacter subsp. michiganensis*	C290	Louws et al., 1998


	BL-Cmm17	Xu et al., 2010
	A226, A300CMM12B	Louws et al., 1998
	08-687, 09-158, 09-159, VF3-1-08, VF6-7-08, 09-176, SM101-09, 09-157, SM287-11, SM288-11, SM610-11, SM611-11, SM614-11, SM615-11	Different clonal groups of Cmm strains isolated from greenhouse tomatoes in USA, Canada, and Guatemala

*Pseudomonas fluorescens*	Wood1	Plant beneficial bacteria, provided by Dr. Brian McSpadden Gardener


*Bacillus subtilis*	GB03	
*Mitsuaria* sp.	H24L5A	
*Lysobacter enzymogenes*	C3	

*Lactobacillus rhamnosus*	LGG	Human gut bacteria, lab collection
*Bifidobacterium animalis*	Bb12	
*Escherichia coli*	Nissle	

### Primary Screen

Primary screening was conducted with Cmm wild-type strain C290, which was originally isolated from tomato in Ohio and characterized as type C by REP-PCR (Louws et al., 1998). Briefly, a fresh bacterial culture was inoculated into 5 ml NBY broth and grown at 28°C with shaking at 200 rpm. After 24 h of incubation, the culture was diluted in NBY broth to an OD_600_ of 0.05 (5 × 10^7^ CFU/ml) using a Genosys 20 spectrophotometer (Thermo Scientific, Rockford, IL, USA). To an aliquot of 100 μl diluted culture in each well in a 96-well plate, 1 μl of compound was added using a slotted pin tool (V and P Scientific, San Diego, CA, USA) for a final concentration of 200 μM. Controls (four replicates/plate) containing 1 μl DMSO, 1 μl chloramphenicol (20 μg/μl), no compound and 100 μl of cell-free media were included in each test plate. Plates were incubated at 28°C with shaking at 200 rpm for 24 h. The end-point OD_595_ was measured using a Sunrise^TM^Tecan kinetic microplate reader (Tecan US, Inc., San Jose, CA, USA). A parameter Z’ to evaluate the quality of the HTP screen was calculated using formula 1 (Zhang et al., 1999). The growth inhibition rate was calculated as described by formula 2. The culture in wells with ≥99% growth inhibition was streaked onto fresh NBY agar, as were the sterility, antibiotic and no compound control wells. Bacterial growth was measured on the plate after 48 h at 28°C. Based on the recovery of Cmm on NBY, the compound was scored as either “static” or “cidal.”

$$Formula\,\left(1\right)\,:Z^{\prime }=1-\left(3\sigma_{c^{+}}+3\sigma_{c^{-}}\right)/|\mu_{c^{+}}-\mu_{c^{-}}|,$$

where σ_c^+^_, σ_c^-^_, μ_c^+^_, and μ_c-_are the standard deviation and average of positive (DMSO amended) and negative controls (chloramphenicol amended).

$$Formula\,\left(2\right)\,:Inhibition\,rate=\left(\mu_{c^{+}}-X\right)/{\left(\mu_{c^{+}}-\mu_{c^{-}}\right)}^{*}100\%,$$

where μ_c^+^_, and μ_c-_ are the average OD of positive and negative controls, X is the OD in well with the small molecule compound.

### Secondary Screen with Selected Compounds

A structural analysis of the primary screen data for 468 hit compounds was conducted. The structural descriptor strings (SMILES) were subsequently converted into ChemDraw structures using ChemDraw for Excel. The compounds were exported to ChemDraw as a SD file using ChemFinder. ChemFinder resulted in the rapid identification of compounds containing the same structural motifs. The ChemDraw files of the hits were manually sorted into structural groups to establish preliminary structure-activity relationships (SARs). Finally, hits were prioritized for secondary screens based on their adherence to Lipinski’s rule of 5 (Lipinski et al., 1997).

Seventy-seven selected compounds were re-ordered from ChemBridge in a 96-well format as a powder. The compounds were dissolved in 100 μl DMSO and stored at -80°C until used. Five tests were carried out to evaluate the sensitivity and specificity of selected compounds, including testing: (1) multiple Cmm strains from different clonal groups as listed in **Table 1**; (2) the minimum inhibitory concentration (MIC) for Cmm growth; (3) the minimum bacteriocidal concentration (MBC) for Cmm (Ling et al., 2015); (4) effects on growth of plant beneficial bacteria and human gut bacteria listed in **Table 1**; and (5) cytotoxicity of the most potent compounds to Caco-2 cells. In addition, ability of Cmm to develop resistance to selected compounds was tested.

Screening for growth inhibition of Cmm strains, plant beneficial bacteria and human gut bacteria was set up similarly to the initial screen using 1 μl of 2 μmol suspensions of each compound. Plates were incubated in a Sunrise^TM^ Tecan microplate reader for kinetic measurement of growth every 15 min for 24 h. Growth curves were analyzed in DB interface software and the effect of each compound on growth was evaluated based on the ratio of area under growth curve (compound/control) as “no significant effect (ratio > 0.5),” “inhibition (ratio ≤ 0.5),” “static (ratio = 0, bacterial growth revived after streak on a fresh NBY plate)” and “cidal (ratio = 0, bacterial growth not revived)” (Wallace et al., 2011).

### Germination and Phytotoxicity Assessment of Selected Compounds

The effect of selected compounds on seed germination and phytotoxicity was evaluated on both tomato and *Arabidopsis*. *Arabidopsis* seeds (cv. Columbia) were surface-sterilized by washing with 70% ethanol-0.05% Triton for 25 min, followed by 100% ethanol for 10 min. Molten 1% water agar was amended with each selected compound at a ratio of 1:100 (1 μl of 2 μmol compound:100 μl water agar) and added to wells of a 96-well plate. *Arabidopsis* seeds were suspended in sterilized water and 5 μl (approximately 10 seeds) was pipetted into each well in a 96-well plate. The germination rate of seed in each well was recorded 5 days later. Similarly, tomato seeds (cv. Tiny Tim) were treated with hot water to eliminate internal and external bacterial phytopathogens (Miller and Ivey, 2005). Five seeds were tested in each well of a 48 well-plate containing 200 μM small molecule amended 500 μl of 1% agar. The seed germination rate in each well was recorded 5 days later.

To determine whether a selected compound was phytotoxic, 1 μl of 2 μmol compound diluted in 100 μl water was applied to 10-days-old tomato seedlings in 96-well library tubes and 10-days-old *Arabidopsis* seedlings in 96-well plates. Death or abnormal growth of seedlings was assessed daily for 5 days. Controls of DMSO (1%), thymol (1.2%), and 2, 4-D (2%) were included in both seed and seedling tests.

### Effect of Compounds on Cmm Infection of Tomato Seedlings

A bioluminescent Cmm strain, BL-Cmm17, was used to monitor the effect of selected small molecules on Cmm infection of tomato seedlings *in vivo* (Xu et al., 2010). Briefly, tomato seeds (cv. OH9242) were infested by soaking in BL-Cmm17 suspension (10^8^ CFU/ml) in a 100-ml sterile beaker. The beaker was placed in a Nucerite Desiccator (Nalge Sybron Corporation, Rochester, NY, USA), and a vacuum was applied for 5 min using an Air Cadet pump (Barnant, Barrington, IL, USA) with a maximum of 18 lb/in^2^ pressure. Seeds treated similarly with sterilized water were used as controls. After inoculation, seeds were air-dried and one seed was placed in a 1.2 ml library tube containing 400 μl of 1% water agar; tubes were placed in wells of a 96-well plate. The selected compound (1 μl of 2 μmol in 50 μl water) was applied to each seed and the plate was incubated at 25°C under 8 h/16 h light/dark conditions. There were three replicate seeds/plate per treatment. Bioluminescence images were taken using an *in vivo* imaging system (IVIS Model 100; PerkinElmer, Waltham, MA, USA) 3 and 8 days later. Eight-days-old seedlings were ground in potassium phosphate buffer and extracts were serially diluted and plated on NBY to assess the presence of Cmm.

### Cytotoxicity of the Most Potent Compounds

Caco-2 cells (human colonic carcinoma) were obtained from the American Type Culture Collection, Rockville, MD, and maintained in growth medium [minimal essential medium (MEM) supplemented with 20% fetal bovine serum (FBS), 1% non-essential amino acid (NEAA, Invitrogen Life Technologies, Grand Island, NY, USA) and with 1 mM sodium pyruvate] at 37°C in a humidified, 5% CO_2_ incubator. Lactate dehydrogenase (LDH) Cytotoxicity assay was performed following the manufacturer’s instructions (LDH Cytotoxicity Assay Kit, Pierce ^TM^, Thermo Scientific, Rockford, IL, USA). Briefly, ∼1.4 × 10^5^ cells were grown in a 96-well tissue culture plate with 150 μl of growth medium and incubated for 24 h at 37°C in a humidified, 5% CO_2_ incubator until a monolayer was completely formed. After three washes with medium without supplements, 1 μl of 2 μmol compound was added to 100 μl of media in each well and incubated for 4 h at 37°C in a humidified, 5% CO_2_ incubator. Fifty microlitre of cell supernatant were collected and LDH was measured using the controls indicated by the kit. Blank controls were used by adding 1 μl of DMSO and values subtracted from the readings.

### Potential for Cmm Acquisition of Resistance to Selected Inhibitory Compounds

Single step and sequential passage resistance assays were performed as described previously with few modifications (Ling et al., 2015). Eleven of the 12 top hit compounds were tested in this experiment and one could not be resynthesized. The MIC for these 11 compounds was determined using concentrations from 100 to 2.5 μM. The MBCs for each of these compounds were determined as described previously (Ling et al., 2015). These data were then used for determination of the lethal and sub-lethal doses for resistance studies.

#### Evaluation of Resistance to Compounds Using Sequential Passage at Sub-lethal Doses

*Clavibacter michiganensis* subsp. *michiganensis* cultures grown in NBY broth medium at 28°C for 24 h were suspended in fresh NBY broth and normalized to an OD_600_ of 0.01 (5 × 10^6^ CFU/ml). One-hundred microliters fresh culture was transferred to duplicate wells of a 96-well microtiter plate each containing 0.75× MIC of a test compound (concentration allowing >50% growth inhibition). Cmm cultured in 20 μg/ml chloramphenicol, 50 μg/ml kanamycin, or 2% DMSO, or non-amended NBY broth were used as controls. The bacteria were incubated at 28°C, 150 rpm, for 24 h in the dark. Following incubation, plates were centrifuged at 2,100 × *g* for 5 min at room temperature. The supernatant was discarded, bacteria were resuspended in 100 μl fresh NBY and transferred into a new 96-well plate containing 0.75× MIC of the same small molecule. This procedure was repeated 14 times. Following 15 passages, bacterial suspensions were assessed for resistance to the test compound by assessing MIC and MBC as noted above.

#### Evaluation of Resistance to Compounds Using a Single Step at a Lethal Dose

Cmm grown in NBY medium at 28°C for 48 h was centrifuged at 4,700 × *g* for 15 min at room temperature. Supernatant was discarded and bacteria were resuspended in sterile water to a concentration of 2 × 10^10^ CFU/ml). Compounds (2× MIC) were mixed with 1 μl of molten NBY agar medium and transferred to duplicate wells of a 24-well culture plate. Agar in the plate was allowed to solidify in the dark. Fifty microlitre of Cmm culture (1 × 10^9^ CFU) was added to test wells, dried in the dark at room temperature, and incubated at 28°C for 5 days in the dark. Bacteria spread on NBY agar with 50 μg/ml kanamycin or NBY agar itself was used as positive and negative controls, respectively. After 5 days of incubation, any colonies that developed were assessed for resistance to the test compounds by determining MIC and the MBC as noted above.

### Data Analysis

To facilitate data analysis, nominal scales on growth inhibition in the secondary screen were transformed to ordinal scores. The compound effect on Cmm growth was scored as “cidal” = 4, “static” = 3, “inhibition” = 2 and “no effect” = 1, and the effect on plant beneficial bacteria and human gut bacteria growth was scored as “cidal” = 1, “static” = 2, “inhibition” = 3 and “no effect” = 4. The compound effect on seed germination of tomato and *Arabidopsis* was scored as “germination rate (GR) = 100%” = 4, “100% > GR ≥ 80%” = 3, “80% > GR ≥ 50%” = 2, “GR < 50%” = 1. The total score of each compound was added up from the specificity, sensitivity and phytotoxicity tests carried in the secondary screen. The cytotoxicity of the most potent compounds was analyzed by one-way analysis of variance with mean separation by a least significant difference test at 5% level of significance in Minitab 16 statistical analysis software.

## Results

### Primary Screen Identified 468 Compounds Inhibitory to Cmm

**FIGURE 1:**
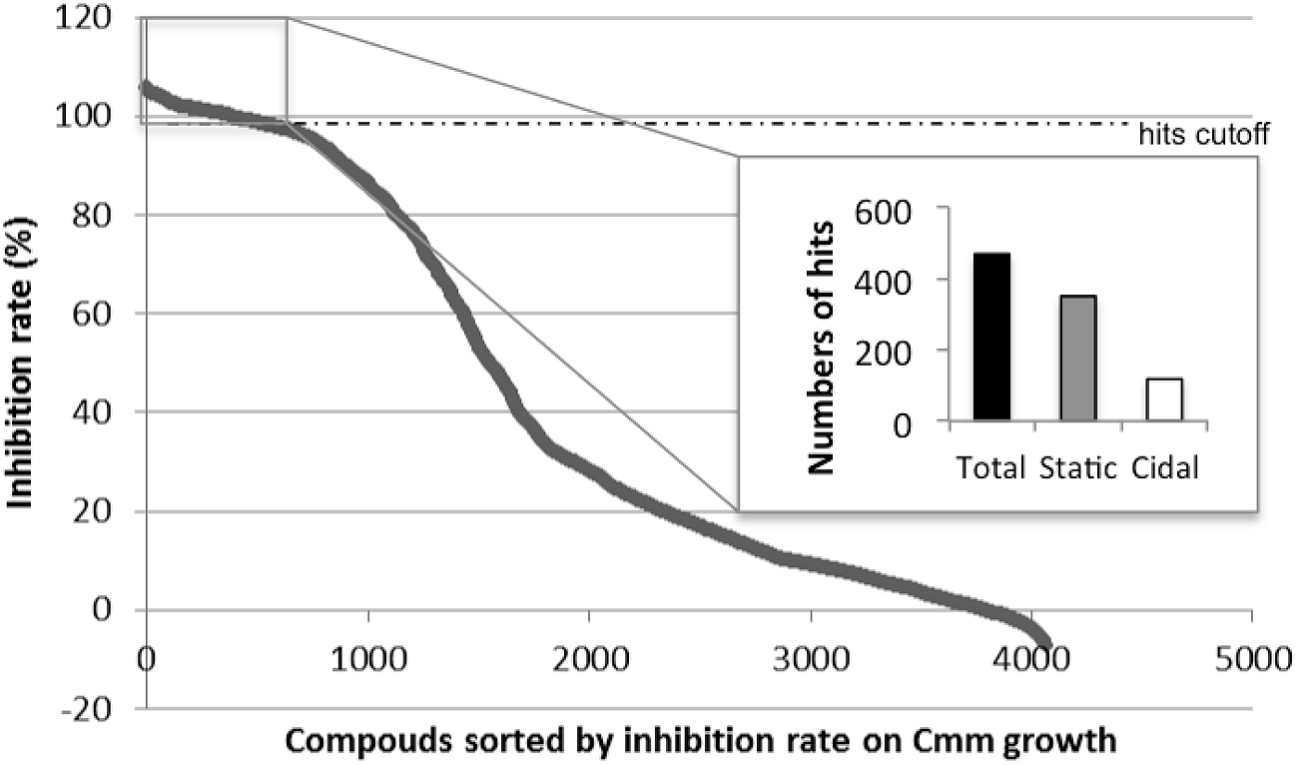
**Initial screen of 4,182 compounds against *Clavibacter michiganensis* subsp. *michiganensis* strain C290 growth.** The 468 small molecules with inhibition rate over 99% were separated based on the effect on *Cmm* (350 statics and 118 cidals) and selected as candidates for further analysis.

A total of 4,182 small molecules were tested in the primary screen against Cmm growth in 96-well plates. As all small molecules were dissolved in DMSO, it was important to confirm whether Cmm growth was affected by DMSO. The supplement of 1 μl DMSO in 100 μl culture did not significantly affect Cmm growth *in vitro* (see Supplementary Figure S1). The initial screen was an evaluation of growth by measuring the end-point OD_595_ value and the average of the statistical parameter Z’-factor was 0.82 (see Supplementary Figure S2). Z’-factor described the signal window and variation within the positive and negative controls and a Z’ value higher than 0.5 was considered a robust HTP assay (Zhang et al., 1999). Compounds exhibiting high inhibition of growth (>99% inhibition) were considered candidates for future evaluations. With this criterion, 468 hits were identified in the primary screen (**Figure 1**). Among these candidates, 350 exhibited a “static” effect, for which Cmm was revived after streaking onto a new NBY plate; and 118 were “cidal” to Cmm growth, in that Cmm was not revived after streaking.

### Compound Prioritization for Further Evaluations

The purpose of this analysis was to identify compounds that were active against the pathogen and may possess novel mechanisms of action that convey selectivity for a specific pathogen. To study the active agents in greater detail, 77 compounds were selected for additional screening. We attempted to ensure that these compounds had acceptable physicochemical properties for further development as potential therapeutic agents by prioritizing those based on: (i) their adherence to Lipinski’s rule of 5 (a measure of the drug-likeness of chemical compounds; Lipinski et al., 1997), (ii) meeting the criteria of the golden triangle analysis, and (iii) absence of obvious reactive functional groups. Additional selection criteria include the ability to rapidly functionalize the molecule through synthetic methods and the novelty of the structure. Based on these criteria, the unique compounds with bactericidal activity have been the primary focus of this effort. For this reason, several of these compounds were included with the compounds selected for additional screening.

### Sensitivity and Specificity of Selected Compounds

**FIGURE 2:**
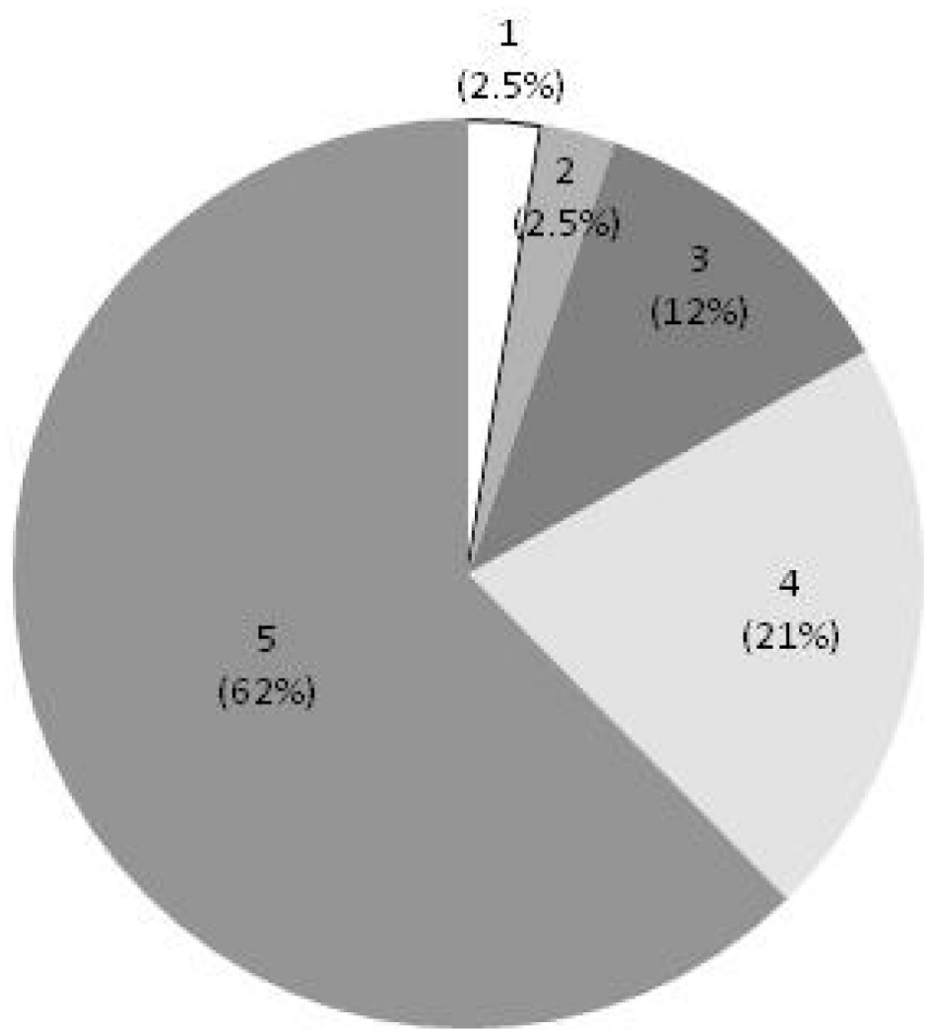
**Effect of selected compounds on multiple *C. michiganensis* subsp. *michiganensis* (Cmm) strains.** Score 5 = cidal to five groups of Cmm strains, 4 = cidal to four groups of Cmm strains, 3 = cidal to three groups of Cmm strains, 2 = cidal to two groups of Cmm strains, 1 = cidal to 1 group of Cmm strains.

In the secondary screen, compounds were evaluated by kinetic measurement of Cmm growth over 24 h instead of end-point measurement used in the primary screen (see Supplementary Figure S3). All the 77 selected compounds showed either a cidal or static effect on the five groups of Cmm strains tested, which confirmed the reproducibility of the results from the primary screen. Forty-eight of the candidates (62%) were cidal to all Cmm strains and 33% were cidal to at least three groups of Cmm strains (**Figure 2**).

**FIGURE 3:**
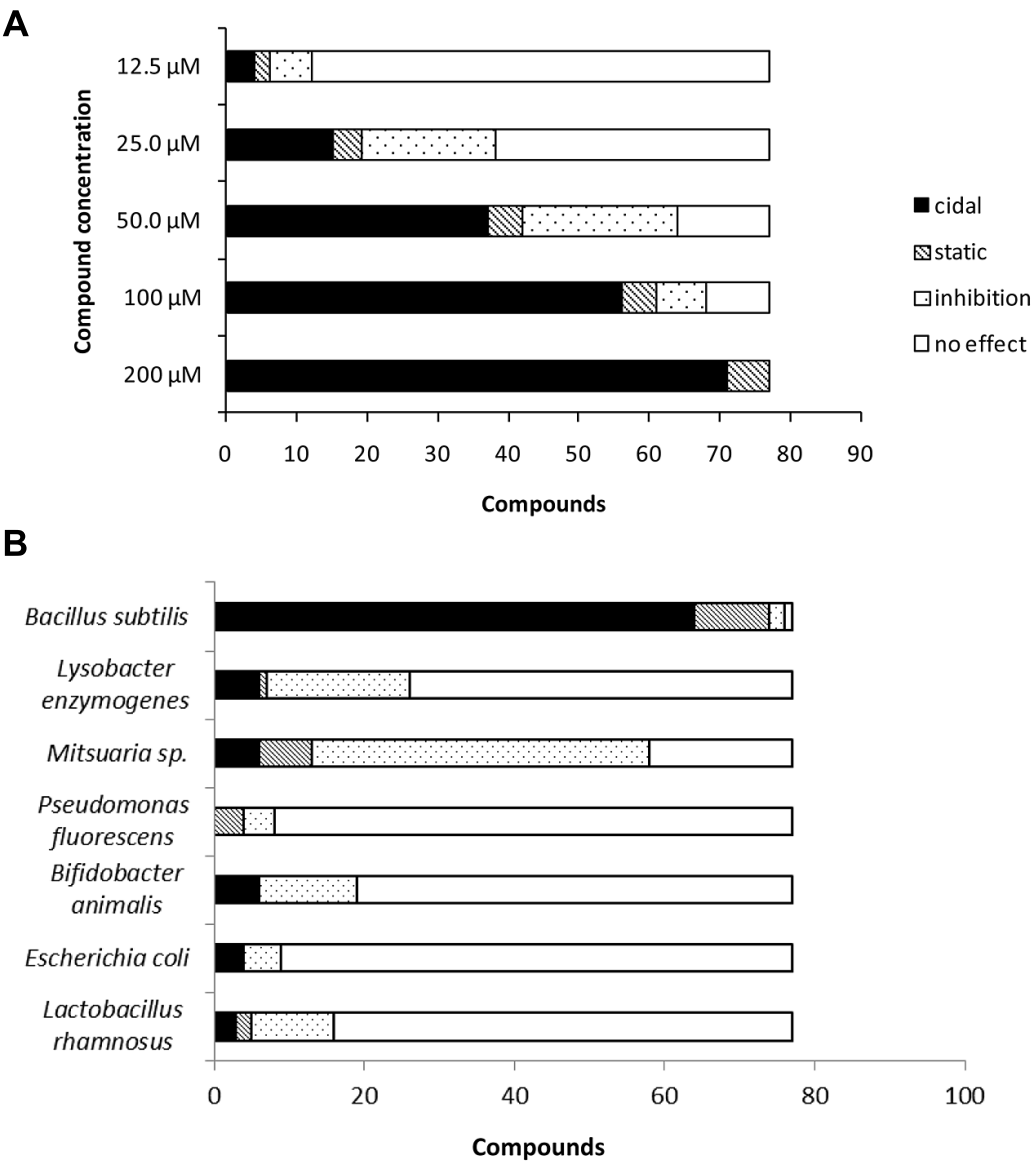
**(A)** Effect of serially diluted compounds on *C. michiganensis* subsp. *michiganensis* growth. **(B)** Effect of selected compounds on growth of plant beneficial bacteria and human gut bacteria.

While testing the compounds for MIC against Cmm, we found that cidality was concentration-dependent; half of compounds were not effective at 25 μM; at 12.5 μM four candidates remained cidal (**Figure 3A**). To assess the specificity of the compounds, they were screened at 200 μM concentration against *Pseudomonas fluorescens* and *Lysobacter enzymogenes* (plant commensal bacteria), and *Bifidobacterium animalis, E. coli*, and *Lactobacillus rhamnosus* (human gut commensal bacteria). Most compounds did not significantly affect the growth of plant and human beneficial bacteria (**Figure 3B**). In contrast, the growth of the plant rhizospheric bacterium *B. subtilis* was completely inhibited by most compounds and the growth of *Mitsuaria* sp. was affected by more than half of the candidates.

### Phytotoxicity of the Selected Compounds

Some compounds reduced the germination of *Arabidopsis* seeds. All non-treated *Arabidopsis* seeds germinated, whereas the rate of germination for seeds treated with DMSO alone was 85%. Forty-one of 77 (53%) compounds had no or little effect on *Arabidopsis* germination (≥80%), 15 candidates moderately reduced germination (50–100%), and 21 candidates reduced germination rates to less than 50% (**Table 2**).

**Table 2:** Effect of selected compounds on *Arabidopsis* and tomato seed germination.

Germination rate (%)	Score	Number of compounds	
		*Arabidopsis*	Tomato
100	4	24	71
80 ≤ GR < 100	3	17	1
50 ≤ GR < 80	2	15	2
<50	1	21	3

The majority of hit compounds (93%) did not affect tomato seed germination compared to untreated and DMSO controls. The germination rate of tomato control seeds, both non-treated and DMSO treated was 100%. Only six compounds reduced germination less than 80%. Compounds applied to seedlings did not cause deformation or death in either *Arabidopsis* or tomato. Together the data on both *Arabidopsis* and tomato seeds suggest that the majority of the compounds are specific to bacteria at the doses tested.

### Efficacy of Selected Compounds in Controlling Cmm on Tomato Seed

**FIGURE 4:**
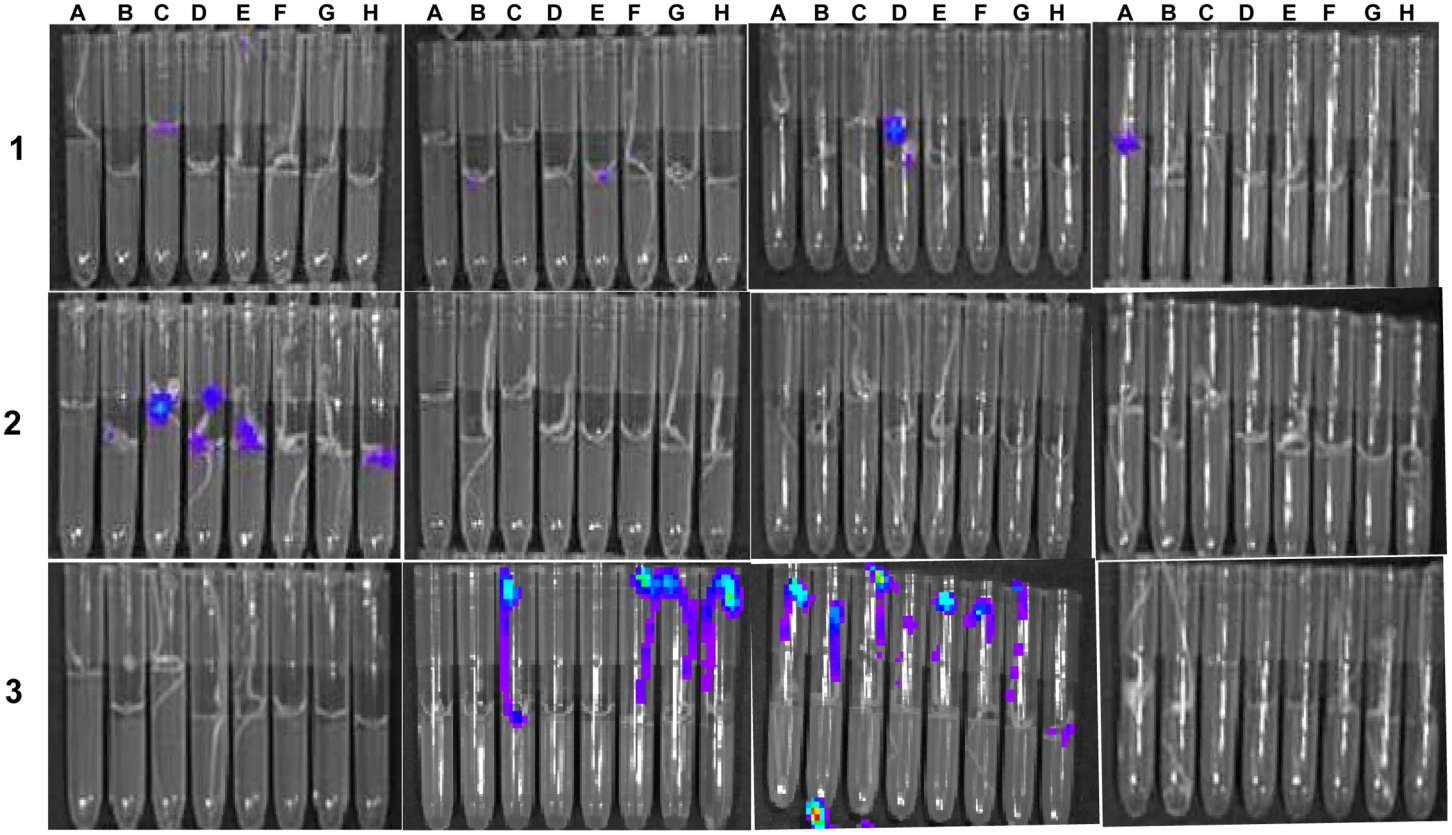
**Bioluminescence imaging of tomato seed infested with *C. michiganensis* subsp. *michiganensis* (Cmm) BL-Cmm17 and treated with selected small molecules in 96-well library tubes.** Image was taken of 8-days-old seedlings with an *in vivo* imaging system. Tubes in rows 1, 2, and 3 contained Cmm-infested seeds treated with small molecules; seeds in row 3, column 2 (F, G, H) were Cmm-infested and treated with DMSO; seeds in row 3, column 3 (A through H) were Cmm-infested and not treated with small molecules; and seeds in row 3, column 4 (A through H) were not non-inoculated with Cmm.

**FIGURE 5:**
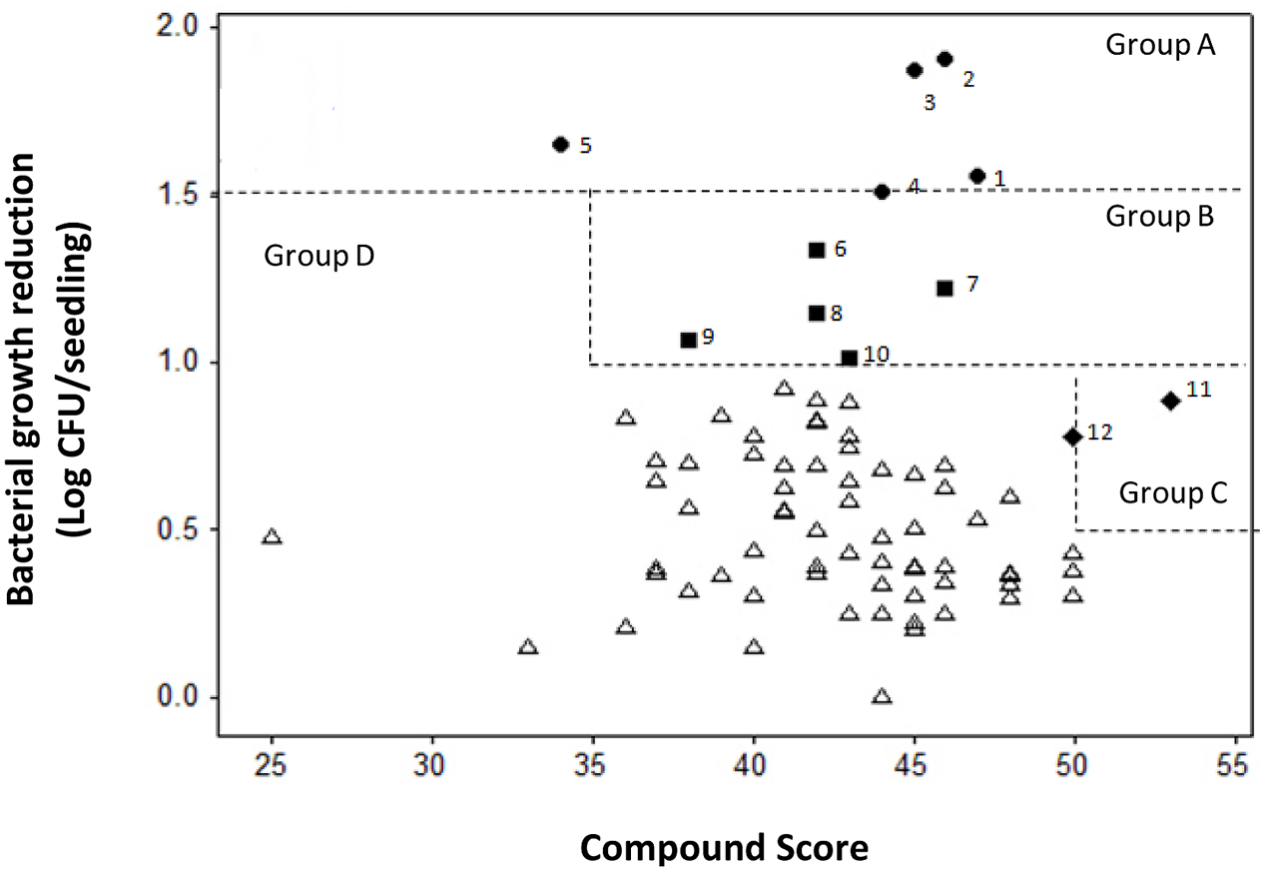
**Scatter plot of compound score versus reduction of bacterial growth in tomato seedlings infected with *C. michiganensis* subsp. *michiganensis* (Cmm).** Compounds were categorized into four groups: Group A included compounds (1–5) with Cmm growth reduction over l.5 log; Group B included compounds (6–10) with Cmm growth reduction over 1.0 log and score over 35; Group C included compounds (11 and 12) with Cmm growth reduction over 0.5 log and score over 50; and Group D included compounds that did not reach the criteria for Groups A, B, or C.

Cmm BL-Cmm17 is a virulent bioluminescent strain carrying *luxCDABE* and is a useful reporter because the strength of luminescent signals is positively correlated with the number of live cells (Xu et al., 2010). Bioluminescent imaging of inoculated tomato seedlings showed that the non-treated, infected tomato seedlings exhibited high luminescent signals compared to Cmm-inoculated seedlings treated with most compounds. Seedlings treated with nine of 77 compounds exhibited high luminescent signals, indicating that these compounds did not inhibit Cmm infection (**Figure 4**). However, half of the compounds reduced the tomato Cmm burden by over 0.5 log CFU; 10 compounds reduced Cmm populations by 1 log or greater (**Figure 5**).

**FIGURE 6:**
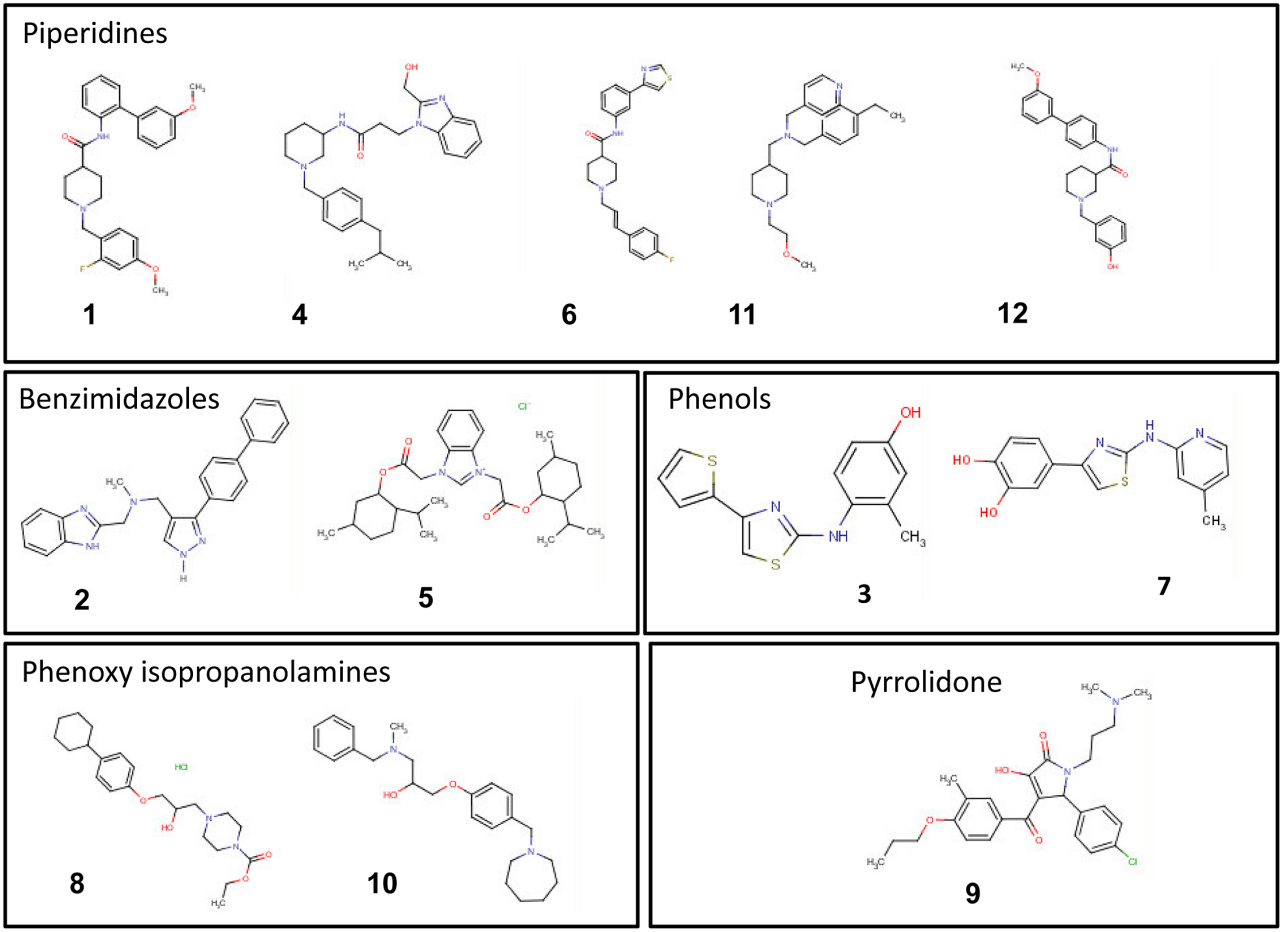
**Chemical structures of the top 12 potent small molecules inhibitory to *C. michiganensis* subsp. *michiganensis* identified in this study**.

The minimum and maximum sum scores for specificity, sensitivity and phytotoxicity in the secondary screen were 14 and 57, whereas the actual score ranged from 25 to 53. A scatter plot of the compound scores versus effect on Cmm seedling infection allowed us to categorize these compounds into four groups (**Figure 5**). The most potent compounds were identified by considering both the effect on reduction of Cmm infection in seedlings and high score in the secondary screens. Thus, compounds 1 to 12 from Groups A, B, and C were considered to have a strong potential for bactericide development. The chemical structures of the 12 compounds falls into five distinct classes: piperidines, benzimidazole, phenols, phenoxy isopropanolamines, and pyrrolidones (**Figure 6**).

### Cytotoxicity of Selected Compounds

**FIGURE 7:**
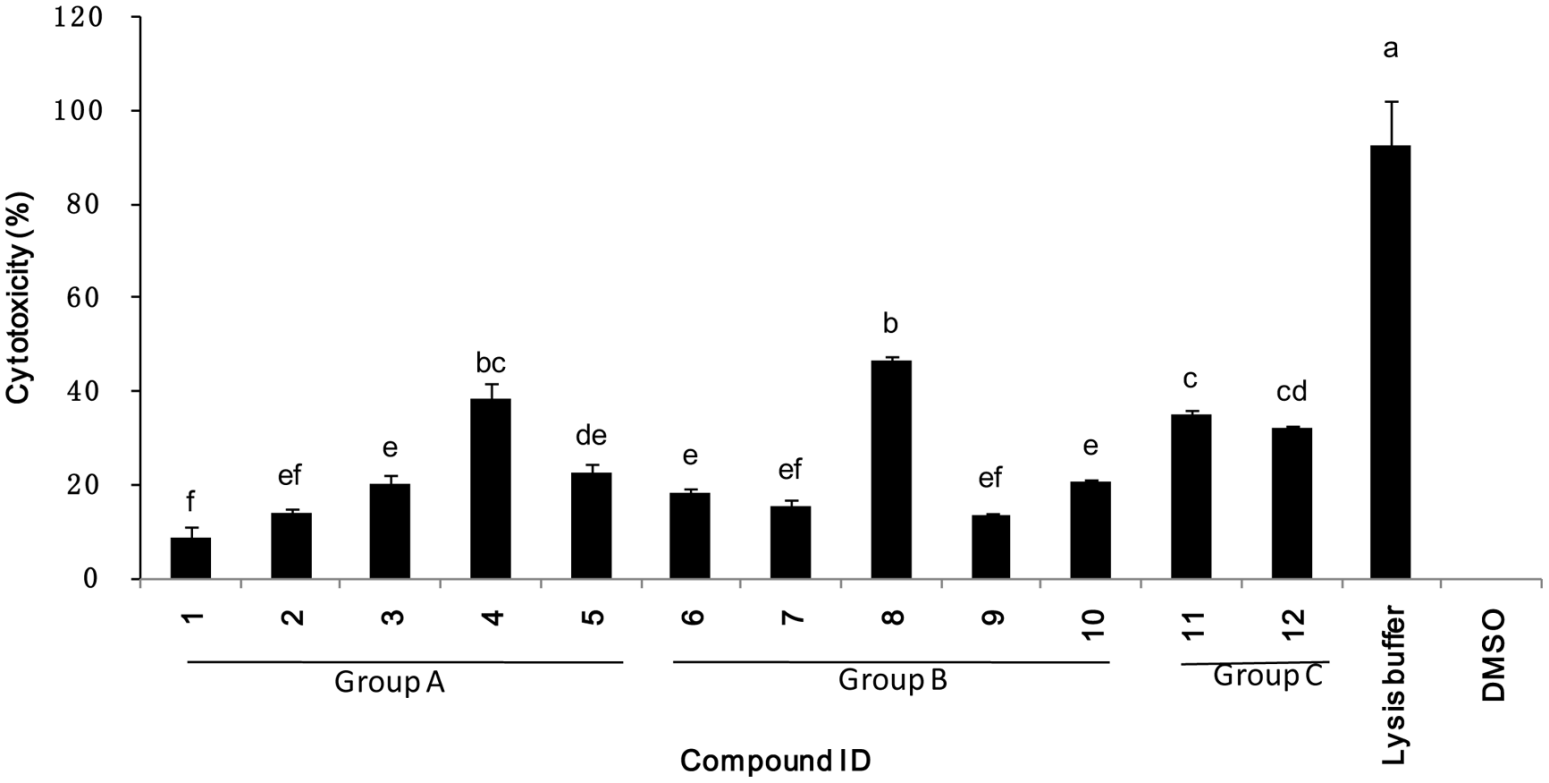
**Percentage mammalian cytotoxicity of the 12 top potent compounds.** Cytotoxicity was assessed using Caco-2 cells exposed to 200 μM of compound for 4 h. Groups A, B, and C refers to compounds classification described in **Figure 5**.

Cytotoxicity was evaluated using cultured mammalian cells to explore the potential future application of these selected compounds on tomato for consumption. The 12 compounds tested showed a range of cytotoxicity of 8.4–46.6% in the cytotoxicity assay (**Figure 7**), compared to the lysis buffer control. These results suggested that the most potent compounds show varying degrees of specificity to bacteria with minor degrees of general toxicity.

### Potential for Cmm Acquisition of Resistance to Selected Inhibitory Compounds

Lethal and sub-lethal doses of eleven candidate compounds were determined (**Table 3**). Cmm was killed or inhibited at concentrations ranging from 5 to 100 μM depending on the small molecule; the MIC and MBC were the same for seven molecules; for three compounds the MBC was onefold higher than the MIC; and for only one compound the MBC was twofold higher than the MIC. After incubation on solid media amended with a lethal dose of the target compound for 5 days, no resistant Cmm colonies were observed for any of the 11 compounds tested (**Table 3**). Following Cmm incubation at sub-lethal doses in liquid media during 15 passages, identical MICs and MBCs were observed for bacteria that grew at the sub-lethal concentration of small molecules (**Table 3**). This suggests that the 11 novel compounds were unlikely to induce resistance in Cmm under the tested conditions; however, for commercial approval of these antimicrobials, more in-depth characterization of potential resistance is warranted.

**Table 3:** Minimum inhibitory concentration (MIC), MBC, and potential induction of resistance in *Cmm* to the 11 selected small molecules.

*N*°	MICs (μM)	MBCs (μM)	Resistant colonies after growth at sub-lethal (0.75X MIC) concentration^a^	Resistant colonies after incubation at lethal (2X MIC) concentration^b^
1	25	25	None	None
2	25	50	None	None
3	50	50	None	None
4	100	100	None	None
5	5	5	None	None
6	50	50	None	None
7	50	50	None	None
8	50	50	None	None
9	50	100	None	None
11	25	100	None	None
12	12.5	25	None	None

*^a^Resistance to compounds was determined after 15 passages in liquid media; ^b^Resistance to compounds was determined after 5 days growth on solid media.*

## Discussion

Here, we report on the results of an HTP-growth screen to identify novel anti-Cmm compounds. We identified 12 such drug-like small molecule compounds that satisfied our pre-defined criteria. The initial goal of the project was to: (i) exploit the pre-selected library to identify hits that completely inhibit the growth of Cmm; (ii) further discover such small molecules that vary in growth inhibition of Cmm and other bacteria using a kinetic study as previously described (Wallace et al., 2011); and (iii) test these small molecules on pathogen infection in the host. By using a library of pre-screened, bioactive compounds, we and others have found that the hit rate in such screens against untested organisms is increased between 4- and 12-fold (Lieberman and Higgins, 2009; Wallace et al., 2011). Specifically we chose the yeast active “yactive” library, and screened approximately 4,200 of the 7,500 compounds (selected from a total of 81,000 compounds) that we have previously shown inhibitory to *Saccharomyces cerevisiae* growth by at least 30%. Consistent with the reported increase in hit-rate with this library, the hit rate in this study was high (11.1%), despite the strict threshold criteria we used to select candidates based on Cmm growth inhibition.

Depending on the screening purpose, different cellular and molecular high throughput screening (HTS) approaches have been developed, and each has different criteria for hit selection. For example, according to a study performed at 12.5 μM, hit rates of 0.024% for *E. coli* and 0.005% for *P. aeruginosa* were observed (De La Fuente et al., 2006). Our study for complete Cmm inhibition by 77 selected compounds (also performed at 12.5 uM), in contrast, showed numbers comparable to classical antibiotics and were 46-fold higher than those reported for *E. coli*.

Compared to the end-point value used in the primary screen, the area under the growth curve calculated by kinetic measurement of growth provides more quantitative data to evaluate compound effect on growth inhibition (Wallace et al., 2011). Therefore, in our secondary screen, we tested the selected 77 compounds on additional Cmm strains as well as other bacteria using a kinetic OD reader. As expected, the majority of compounds showed cidal effect on growth of multiple Cmm strains, but some compounds were static rather than cidal to diverse strains. Since the Cmm strains were collected from different geographic locations and also presented different DNA fingerprint profiles, their compound sensitivity is likely due to their genetic diversity. Interestingly, testing the compounds on other plant beneficial bacteria and human gut bacteria showed that most compounds were cidal against the Gram-positive bacterium *B. subtilis*, but had little or no effect on the Gram-negative bacteria tested. Based on this observation, we suggest that the compounds tested in the secondary screen may disrupt the cell membrane structure, cell wall synthesis or metabolic activity specific to Gram-positive bacteria.

The cell-based HTP screens rely on bacterial growth inhibition, hence they will miss virulence genes that when inhibited do not show a growth defect. However, our rapid, high-throughput, *in vivo* imaging assessment of Cmm infection in tomato may provide a complementary way to identifying small molecules that influence virulence genes or induce plant defenses.

The top 12 drug-like compounds populate five distinct classes (**Figure 6**). The benzimidazole-containing compound carbendazim is a well-known fungicide widely used in agricultural production (Zikos et al., 2015). In addition, recently the benzimidazole class of compounds have been shown to inhibit a G+ bacterium, *Staphylococcus aureus* by targeting DNA gyraseB enzyme (Janupally et al., 2014). We have found two benzimidazole compounds (2 and 5) with specific anti-Cmm activity. In particular, compound #5 with a lower score in the secondary screen exhibited a broader antibacterial spectrum compared with other 11 compounds in this study (data not shown). A few phenolic compounds have been studied for their antimicrobial activity and two natural phenols were less effective against Gram-negative bacteria than Gram-positive bacteria (King et al., 1972). In addition, a phenol drug was found to inhibit *B. subtilis* growth by interfering with cell wall synthesis (Shimi et al., 1976). Consistent with this, we identified two phenol compounds, #3 and #7, which were cidal to both Cmm and *B. subtilis*, but not to the Gram-negative bacteria tested. Hence, we suspect that these phenols have a similar mode of action. Almost half of the 12 top potent compounds belong to piperidines, however, there are very few reports on this class of compounds. A recent study claimed piperidines to possess antimicrobial effects (Patel et al., 2012). Only one of the top compounds, #9, fell into pyrrolidone class, but several previous studies have described pyrrolidone derivatives against human bacterial pathogens (Phaechamud et al., 2012; Sathiyanarayanan et al., 2014). Compounds #8 and #10 were the first phenoxy isopropanolamines reported to have antibacterial activity.

In summary, we have identified 12 anti-Cmm drug-like compounds and future work on increasing the efficacy of the compounds by downstream modification, target identification and biologically active functional groups must be explored.

## Conflict of Interest Statement

The authors declare that the research was conducted in the absence of any commercial or financial relationships that could be construed as a potential conflict of interest.
