# Supplementary material for: Discovery of novel small molecule modulators of Clavibacter michiganensis subsp. michiganensis
Source: Front Microbiol. 2015 Oct 19;6:1127. doi: 10.3389/fmicb.2015.01127 (PMC4609890; doi:10.3389/fmicb.2015.01127)
Supplement: Supplementary file 1 [file Data_Sheet_1.DOCX]

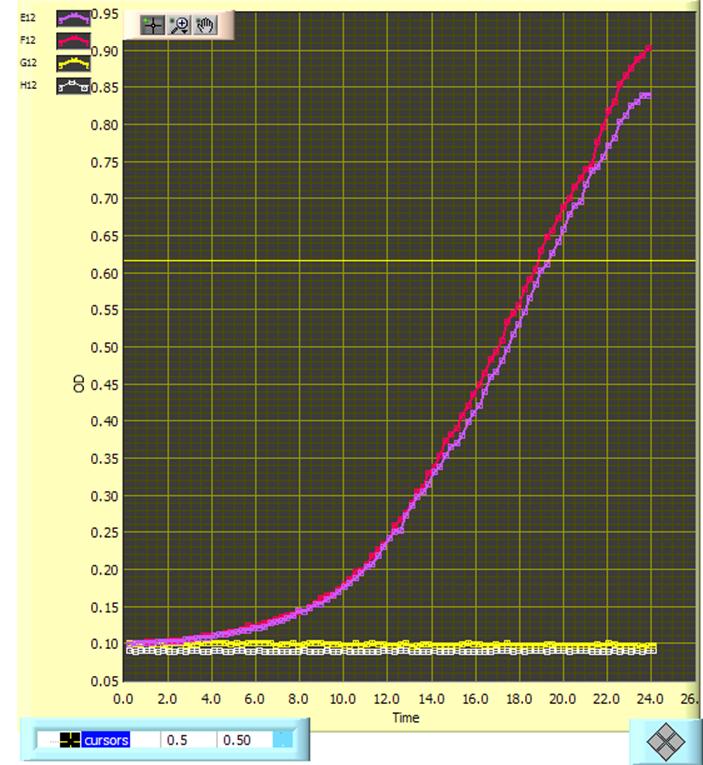

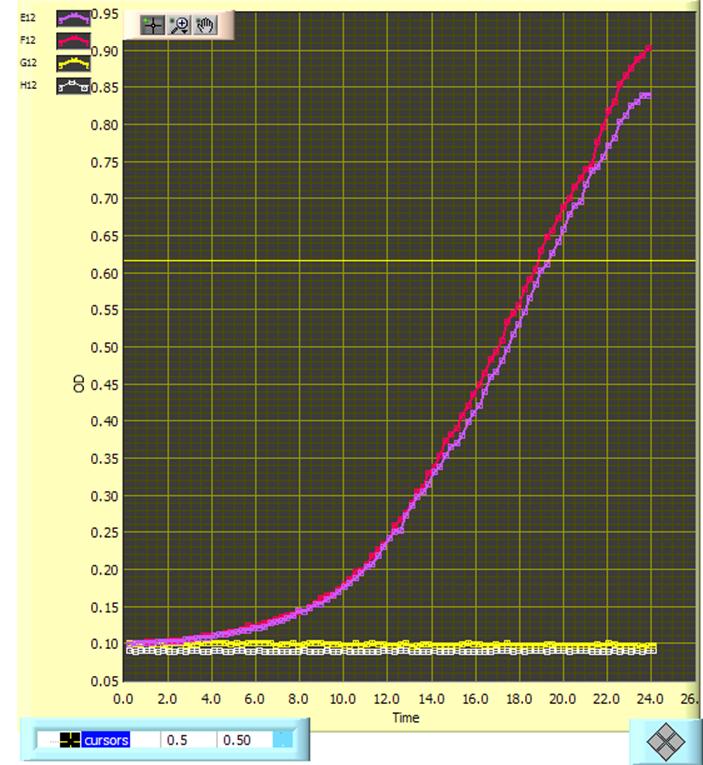


**OD_595_**

**Hrs**

| DMSO |
| --- |
| Control (without compoud) |
| Chloramphenicol |
| NBY medium |

**Supplementary Figure 1 | Growth of *Clavibacter michiganensis* subsp*. michiganensis* strain C290 in 96-well plates was not significantly affected by DMSO.** Data acquired every 15 min over 24 hours.

**Hrs**

**Z’ score**

**Plate number**

**Supplementary Figure** **2 | The statistical parameter Z’-factor for individual library compound plates screened.** The cell-based growth primary screen in this study had an average Z’=0.82. A Z’ value >0.5 and was considered acceptable.


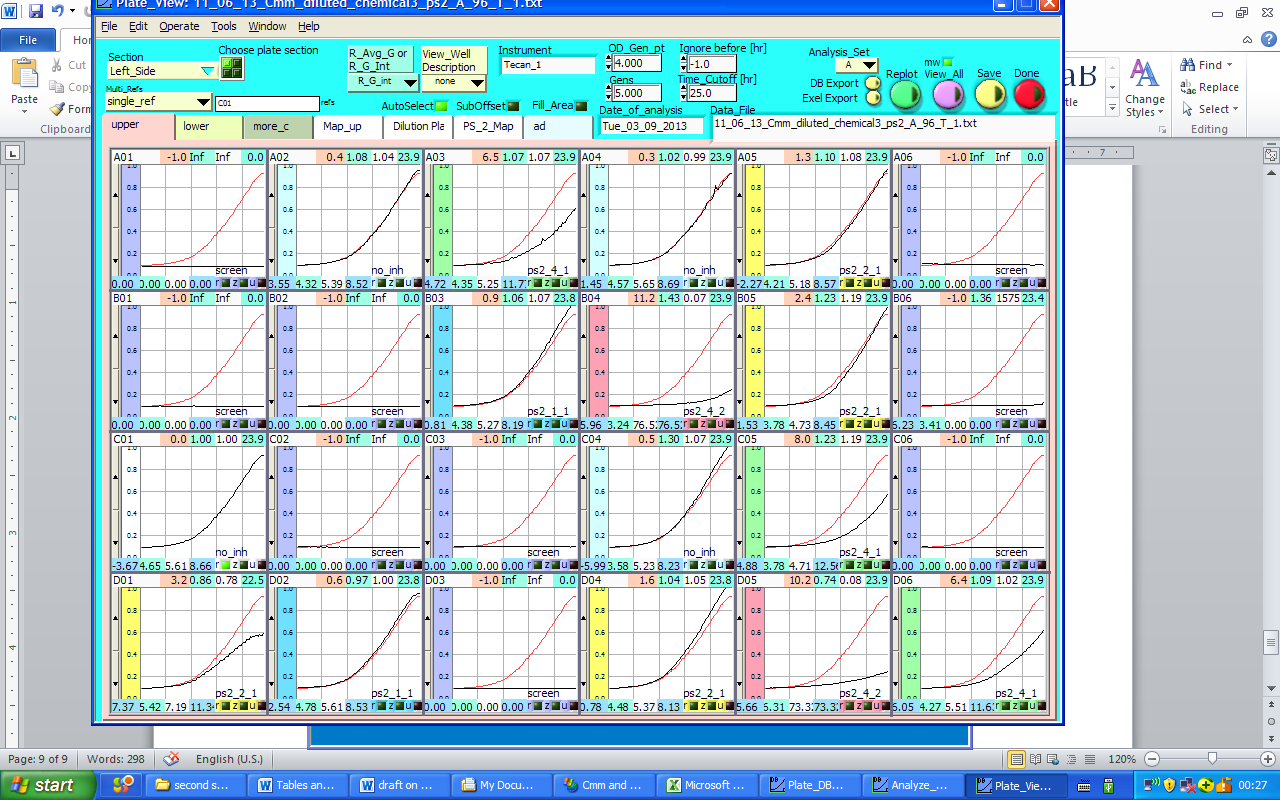


**Supplementary Figure 3 | Yeast Grower (YG) software DB interface analysis of growth curves of *Clavibacter michiganensis* subsp*. michiganensis* strain C290 in a 96-well plate (figure shows part of the plate and is a representative of kinetic growth inhibition of Cmm monitored using Sunrise Tecan automated reader for the selected 77 compounds).** Well A01 was the blank control with NBY broth, B01was the control with chloramphenicol, and C01 was the control without compound and A02, B02, C02, A03, B03, C03 were test wells with small molecules. The growth curve of C01 was setup as a reference curve (black in C01and red in other wells) to compare the difference of growth.
